# Supplementary material for: Causes and consequences of sex-chromosome turnovers in Diptera
Source: Evol Lett. 2026 Mar 12;10(3):253–68. doi: 10.1093/evlett/qrag003 (PMC13231274; doi:10.1093/evlett/qrag003)
Supplement: qrag003_Supplemental_Files [file qrag003_supplemental_files.zip › Supplementary_Datasets.zip!/Supplementary_Datasets/Dataset_2_Graphical_representation_outgroups/Bombyliidae_Villa_cingulata_chromosomes.pdf]

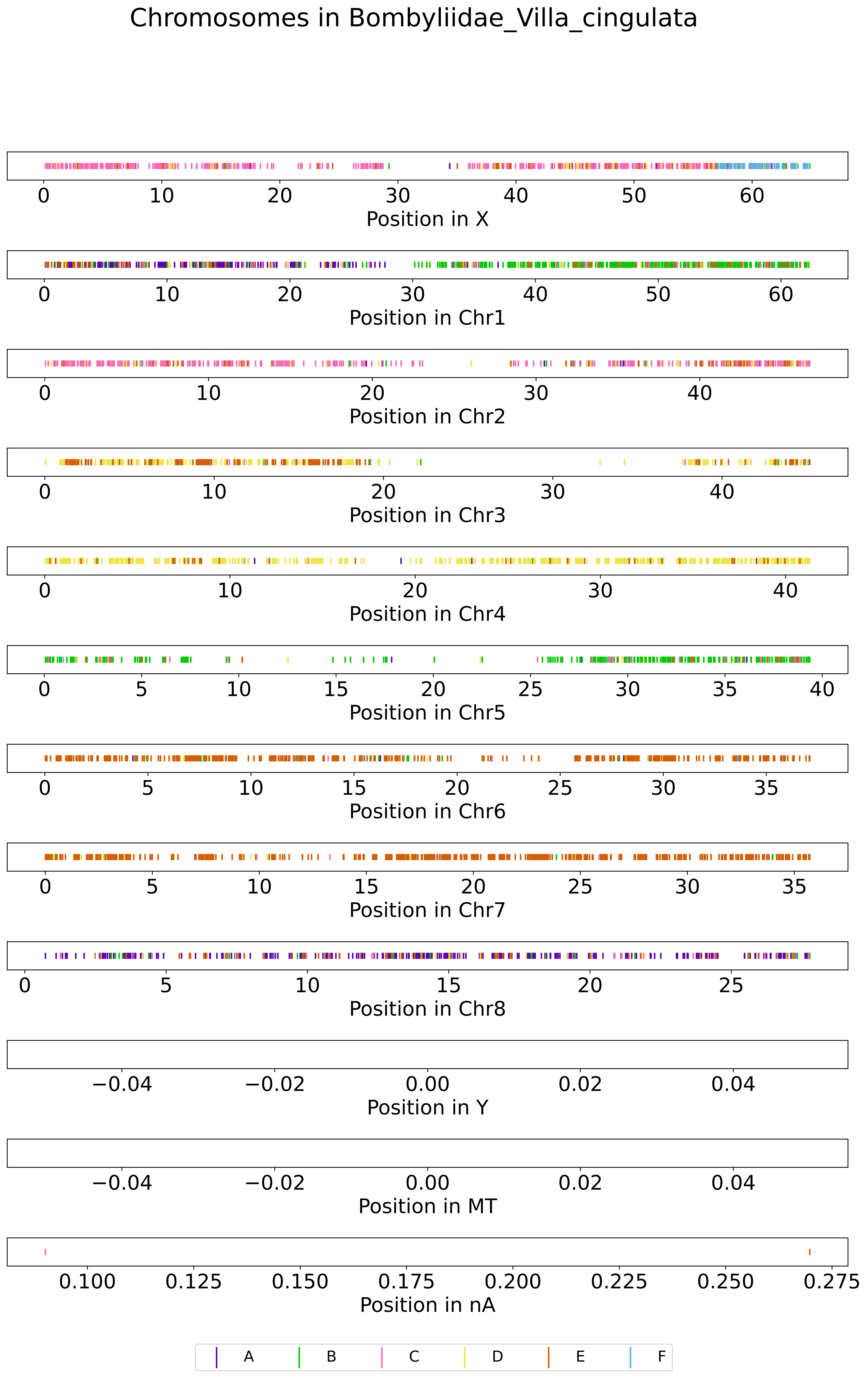
Chromosomes in Bombyliidae_Villa_cingulata
0 10 20 30 40 50 60
Position in X
0 10 20 30 40 50 60
Position in Chr1
0 10 20 30 40
Position in Chr2
0 10 20 30 40
Position in Chr3
0 10 20 30 40
Position in Chr4
0 5 10 15 20 25 30 35 40
Position in Chr5
0 5 10 15 20 25 30 35
Position in Chr6
0 5 10 15 20 25 30 35
Position in Chr7
0 5 10 15 20 25
Position in Chr8
−0.04 −0.02 0.00 0.02 0.04
Position in Y
−0.04 −0.02 0.00 0.02 0.04
Position in MT
0.100 0.125 0.150 0.175 0.200 0.225 0.250 0.275
Position in nA
A B C D E F
